# HD-EEG Based Classification of Motor-Imagery Related Activity in Patients With Spinal Cord Injury

**DOI:** 10.3389/fneur.2018.00955

**Published:** 2018-11-19

**Authors:** Yvonne Höller, Aljoscha Thomschewski, Andreas Uhl, Arne C. Bathke, Raffaele Nardone, Stefan Leis, Eugen Trinka, Peter Höller

**Affiliations:** ^1^Department of Neurology, Christian Doppler Medical Centre and Centre for Cognitive Neuroscience, Paracelsus Medical University of Salzburg, Salzburg, Austria; ^2^Spinal Cord Injury and Tissue Regeneration Center, Paracelsus Medical University of Salzburg, Salzburg, Austria; ^3^Department of Computer Sciences, Paris-Lodron University of Salzburg, Salzburg, Austria; ^4^Department of Mathematics, Paris-Lodron University of Salzburg, Salzburg, Austria; ^5^Department of Neurology, Franz Tappeiner Hospital, Merano, Italy

**Keywords:** spinal cord injury, HD-EEG, connectivity, motor imagery, BCI, ffDTF, FFT

## Abstract

Brain computer interfaces (BCIs) are thought to revolutionize rehabilitation after SCI, e.g., by controlling neuroprostheses, exoskeletons, functional electrical stimulation, or a combination of these components. However, most BCI research was performed in healthy volunteers and it is unknown whether these results can be translated to patients with spinal cord injury because of neuroplasticity. We sought to examine whether high-density EEG (HD-EEG) could improve the performance of motor-imagery classification in patients with SCI. We recorded HD-EEG with 256 channels in 22 healthy controls and 7 patients with 14 recordings (4 patients had more than one recording) in an event related design. Participants were instructed acoustically to either imagine, execute, or observe foot and hand movements, or to rest. We calculated Fast Fourier Transform (FFT) and full frequency directed transfer function (ffDTF) for each condition and classified conditions pairwise with support vector machines when using only 2 channels over the sensorimotor area, full 10-20 montage, high-density montage of the sensorimotor cortex, and full HD-montage. Classification accuracies were comparable between patients and controls, with an advantage for controls for classifications that involved the foot movement condition. Full montages led to better results for both groups (*p* < 0.001), and classification accuracies were higher for FFT than for ffDTF (*p* < 0.001), for which the feature vector might be too long. However, full-montage 10–20 montage was comparable to high-density configurations. Motor-imagery driven control of neuroprostheses or BCI systems may perform as well in patients as in healthy volunteers with adequate technical configuration. We suggest the use of a whole-head montage and analysis of a broad frequency range.

## 1. Introduction

Applications of Brain computer interfaces (BCIs) for neuroprostheses and neurorehabilitation are subject to intensive research (1–3). Potential applications include therapy of neuropathic pain (4), improving reach-to-grasp performance (5), maintaining access to the supplementary motor areas even years after injury (6), cooperation with robotic agents (7–9), and neuroprosthetics, including also electric stimulation with closed-loop control (10–16), among others. High interest of patients in adopting such techniques (17, 18) encourages researchers to put these potential neurorehabilitative measures on a fast-track of development.

The most user-friendly and applicable technology for signal acquisition within BCIs is still the EEG (19). While non-invasive solutions offer wide applicability, neural interfaces with intracortical microelectrodes are believed to provide the most useful control signals for advanced BCIs (20). Recent advances in the field rise the hope that chronic electrode implantation might become a viable solution 1 day (20, 21). For instance, chronic tungsten multielectrode implants deliver reliable signals for up to 6 months after implantation (22).

However, the EEG of patients is considerably different from signals recorded in healthy subjects. Typical applications for brain computer interfaces are conditions of spinal cord injury (SCI) or stroke. Stroke is one of the most devastating conditions related to body paralysis, and it was shown that BCIs can contribute significantly to the rehabilitation of stroke patients (2, 23–25). While stroke is a condition with acute central damage, SCI-related changes develop slowly in the sense of an ongoing neuroplasticity that reflects the loss of afferent feedback from the detached limbs (26). As a consequence, the applicability of BCIs developed with data from healthy subjects to patients with SCI or other pathological changes such as stroke, amyotrophic lateral sclerosis, or locked-in syndrome is highly questionable (27, 28). Indeed, Müller-Putz et al. (29) presented a BCI based on the EEG that worked with a classification accuracy of on average 85.1% in healthy, but 66.1% in patients with SCI. At this point we must recognize that the research results obtained from healthy subjects cannot be transferred easily to patients.

There are several reasons why BCIs developed with healthy subjects cannot be applied to patients with SCI. Neuroplastic changes (30, 31) represent a challenge. Animal models demonstrated that reinforcement learning interfaces could be a good solution in order to address the neuroplastic changes (32). Moreover, high inter- and intraindividual variance (33) represents a technical challenge. The inter- and intraindividual variance is high, but only a few studies presented specific countermeasures such as individual localization (19) or adaptation of imagery tasks (33). For example, movement-related EEG potentials show abnormal patterns in patients with SCI (34, 35).

Nevertheless, most motor-BCI systems are based on modulations of activity in specific frequency bands. BCI-users can be trained to adequately modulate their μ- or β-power in order to control devices. In one of the earliest studies, Pfurtscheller et al. (36) trained a tetraplegic patient over months until he was able to control a hand orthesis by motor imagery. Instead of using a prostheses, Pfurtscheller et al. (37) translated β-signals into functional electrical stimulation, so that a tetraplegic patient could control his paralyzed hands to grasp a cylinder by foot movement imagery. The β-burst was detected online and this signal was then used in order to trigger the functional electrical stimulation. Müller-Putz et al. (38) trained a patient to generate EEG-power decreases by the imagination of movements of his paralyzed left hand. These patterns were classified by the BCI and were used to control a neuroprosthesis for grasping actions. By use of a motor-imagery triggered BCI that partly controlled a hybrid system of functional electrical stimulation, a patient with cervical spinal cord injury could transfer objects with grasp-and-release movements with an accuracy of up to 93% (39).

In addition to these sophisticated systems, newer studies reported a low but still above-chance accuracy of BCIs in patients with SCI even with very simple devices such as the Emotiv EEG system (40). Indeed, it was assumed that the location of electrodes and the coverage of the motor cortex might be crucial for the user in order to obtain reasonable control over the BCI (19). It should be considered whether the fine-grained spatial solution of high-density EEG could improve the performance of such a system. In addition, it is a valid question whether the motor cortex is the only source of informative signals. Channels recording the activity from frontal brain areas could be used in order to capture brain activity related to the planning of the movement.

Moreover, it was suggested that phase synchronization plays an important role in decoding movement from the EEG (41). Gentili et al. (42) reported that a linear decrease of phase synchronization (in terms of coherence and phase locking value) occurred during movement planning and execution. Daly et al. (43) obtained high classification accuracies based on empirical mode decomposition phase locking and derived mean clustering coefficients as complex network metrics extracted from synthetic EEG and a real-EEG with imagined and real finger tapping in 22 young, healthy subjects. As a side note, connectivity measures were also found to distinguish various other cognitive tasks from each other (44). It was suggested that different approaches of spatial coverage are advantageous for motor imagery-based BCI, such as Laplacian filtering (45) or sparse common spatial pattern algorithm (46). An ideal BCI that meets the special needs of patients with SCI, that is, (i) alterations in localization of the imagery-related activity, (ii) poor motor imagery ability but preserved desire/plan to move, could therefore be built using HD-EEG and whole-head coverage in order to capture the most responsive (directed) networks of the brain.

Wang et al. used a local coverage of the motor cortex with electrocorticography and achieved a success rate of 87% in a patient with tetraplegia (47). Magnetoencephlographic signals were used efficiently in order to control a grasping agent in a BCI for patients with SCI (48). However, again, only sensors from the sensorimotor area were used. It would be of interest to examine the systems performance with more sensors, covering the whole cortex.

In the present study, we aim to examine the role of spatial sampling for BCIs in healthy participants and patients. Therefore, we aim to compare the detectability of motor imagery in low- and high density EEG, covering only the motor cortex or the whole head, in patients with SCI vs. healthy controls. Based on previous work we want to replicate the finding that in healthy controls low-density EEG with coverage of the central regions, that is, the motor cortex, allows to achieve good BCI performance with an accuracy well above 85%, while in patients with SCI the accuracy is much lower (29). Consequently, we hypothesize that in patients with SCI the use of whole-head recordings as well as HD-EEG might be more informative and increase the accuracy when classifying brain signals of imagined movement.

## 2. Materials and methods

### 2.1. Ethics

This study was carried out in accordance with the recommendations of Good Clinical Practice. All subjects gave written informed consent in accordance with the Declaration of Helsinki. The protocol was approved by the Ethics Commission Salzburg (Ethikkommission Land Salzburg; approval number 1541).

### 2.2. Subjects

We recruited a total sample of 29 participants for the study at the Department of Neurology, Paracelsus Medical University Salzburg, Austria. A subgroup of 22 healthy participants was recruited via e-mail amongst the students of the Paracelsus Medical University and the Paris-Lodron University, both located in Salzburg. The 7 patients were recruited among the patients of the Department as well as via wheelchair-sport communities. In 4 patients, the experiment was conducted repeatedly with a time between recordings of at least 1 week. All sessions were included in the presented analysis.

### 2.3. Procedure and setting

All participants completed a paper survey on demographic characteristics and the paper and pencil version of the motor imagery questionnaire (MIQ-RS). The MIQ-RS is a questionnaire containing 14 items that aims to assess the ability to perform motor imagery (49). These data were not further processed for the present study but were part of another project. Patients were additionally examined by a medical doctor before the beginning of the study. After that, the HD-EEG recordings were conducted. The task was preceded by a resting state condition which lasted for 2–3 min. Next, the patients got acoustic instructions followed by a training session, including one repetition of each condition. Then, the actual task started (see section 2.4). During all conditions, including the initial resting condition, participants were asked to keep their eyes open and to look at the videos or a fixation cross on a screen. Participants were seated in front of a 11-inch monitor with an approximate distance of 50 cm. All participants had normal or corrected to normal vision (glasses or contact lenses). All sounds were presented by the sound system of the presentation computer at the same volume for each participants.

The resting condition, instruction, training and task lasted in total about 39 min.

### 2.4. Task

There were 7 conditions in the task:

- **2 movement conditions:** Participants were asked to move either the hand or the foot.
- **2 imagery conditions:** Patients were asked to imagine the movement of the hand or the foot.
- **2 observation conditions:** Patients watched a movie showing movement of a real hand or a real foot.
- **1 resting condition:** In the resting condition patients were instructed not to move.

The movement of the hand was a repetitive clenching of the right hand at 1 Hz with 6 repetitions. The movement of the foot was a repetitive tapping of the right foot, again at 1 Hz with 6 repetitions. We did not repeat the same conditions with the left hand or foot, because the overall duration of the experiment took already very long with 25 trials in 7 conditions and 6 s duration per trial.

All of the 7 conditions were acoustically accompanied by 6 repetitions at 1 Hz of two alternating sounds (one alternation per second, i.e., one sound each 0.5 s), which should ensure performance at the required pace. An acoustic stimulation is a better pace-maker than a visual stimulus (50). This cue inserts additional activation e.g., of auditory type, but this activation is the same across all 7 conditions, including the resting condition. Each condition was preceded by an acoustic instruction that lasted 3 s, followed by a pause of 1 s and a startle sound, indicating the begin of the actual condition. All of the 7 conditions were repeated in 25 trials each, in a randomly intermixed order. All trials were separated by inter-trial intervals of 1 s.

**Figure 1:**
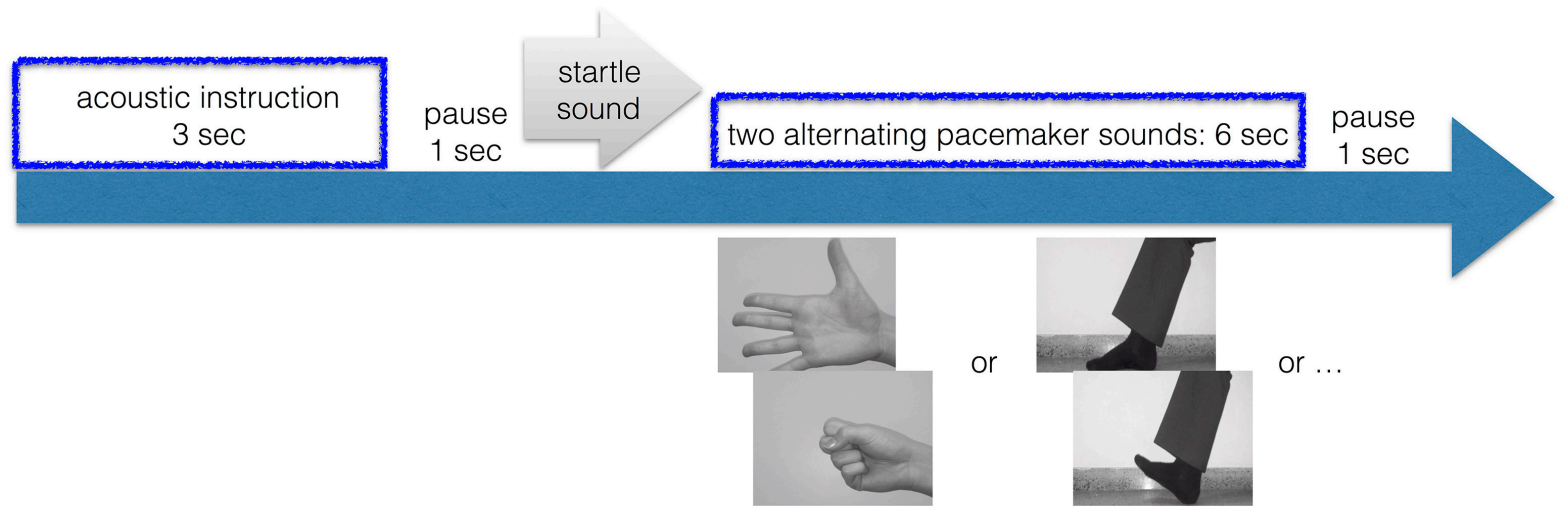
Trial. Time-line of one trial.

Figure 1 illustrates the task.

### 2.5. Data registration

EEG was recorded in a quiet room. We used a 256-channel HydroCell geodesic sensor net and a GES 300 amplifier (Electrical Geodesic Inc., EGI, Eugene, OR). The sampling rate was 250 Hz (4 KHz antialiasing filter). Electrode Cz served as reference. Indeed, Cz is the prime location for foot movement response, so that the signal is emphasized over all electrode positions, and the neighboring central electrodes show up as a bipolar channel with Cz. EGI's NetStation 4.5.6 software was used for data recording. Impedances were kept below 75 kΩ according to recommendations of EGI and the literature (51, 52).

### 2.6. Data preparation

Data was pre-processed with EGIs NetStation 4.5.6 software. Preprocessing included filtering and segmenting of the data. A high-pass IIR filter from 1 Hz and an additional notch filter for removal of line noise (50 Hz) was applied. No further artifact correction was carried out, all trials were included in the further analysis. Most artifacts that were observed were eye blinking artifacts over frontopolar regions, which are unlikely to be linked to a special condition. However, since channels in the face and the neck were those that contained most artifacts and data quality was generally lower in these regions, these channels were excluded.

The data was then segmented into 6,000 ms segments for each participant and each trial, in order to capture the time from onset of the pace-making tone until the end, which should represent a segment including six repetitions of the movement performance, imagery, or observation of the movement. Thus, the segments lasted from the end of the starting sound until the end of the pace-making tone. All conditions were accompanied by the pace-making sound, so that a differentiation by means of the acoustic response should not be possible. In order to have the rest condition as similar as possible to the other conditions, we designed it so that it was preceded by an acoustic instruction and it contained also the alerting stimulus and the pace-making sound. However, the participants were instructed to not move. This should ensure that verbal triggering of activation of the motor cortex is the same across all conditions. The preprocessed data was exported to MATLAB data format^Ⓡ^ (release R2017, The Mathworks, Massachusetts, USA). All further steps were done in MATLAB. First, as a measure of data augmentation, the 6,000 ms segments were additionally segmented into 6 segments of 1,000 ms each, in order to increase the size of the sample. The motivation for this step was that a larger sample size is needed for training the classifier.

### 2.7. Feature extraction

The extraction of features was performed for each of the participants, and for each segment. For each segment, we estimated two features; ffDTF and the power spectrum, estimated as the Fast Fourier Transform (FFT).

Directed transfer function represents information that flows from one region to another over many possible alternative pathways (53). The difference between the directed transfer function and the ffDTF (54) is that the directed transfer function is normalized by the total frequency content of the considered frequency band, while the ffDTF is normalized with respect to all the frequencies in the predefined frequency interval. As such, the ffDTF emphasizes those frequencies which contribute the most to the power of the signal (55). It was shown that the ffDTF is superior over other measures derived from the multivariate autoregressive model in classifying hand vs. foot motor imagery in 14 healthy subjects (56). In a recent publication we could show that the ffDTF is not only highly reliable in general, its reliability is also very robust against artifacts (57), which was highly desirable since exclusion of artifacts was impracticable in the present study due to the low number of trials. In order to estimate the multivariate autoregressive model from which ffDTF can be derived, we used partial correlation estimation with unbiased covariance estimates (58), which was found to be the most accurate estimation method according to Schlögl (59). The model is then transformed from the time-domain into the *z*-domain and the *f*-domain, which yields two transfer functions, accordingly. The multivariate parameters in the frequency domain that can be derived from these transfer functions were computed at 1 Hz frequency steps between 1 and 48 Hz.

**Figure 2:**
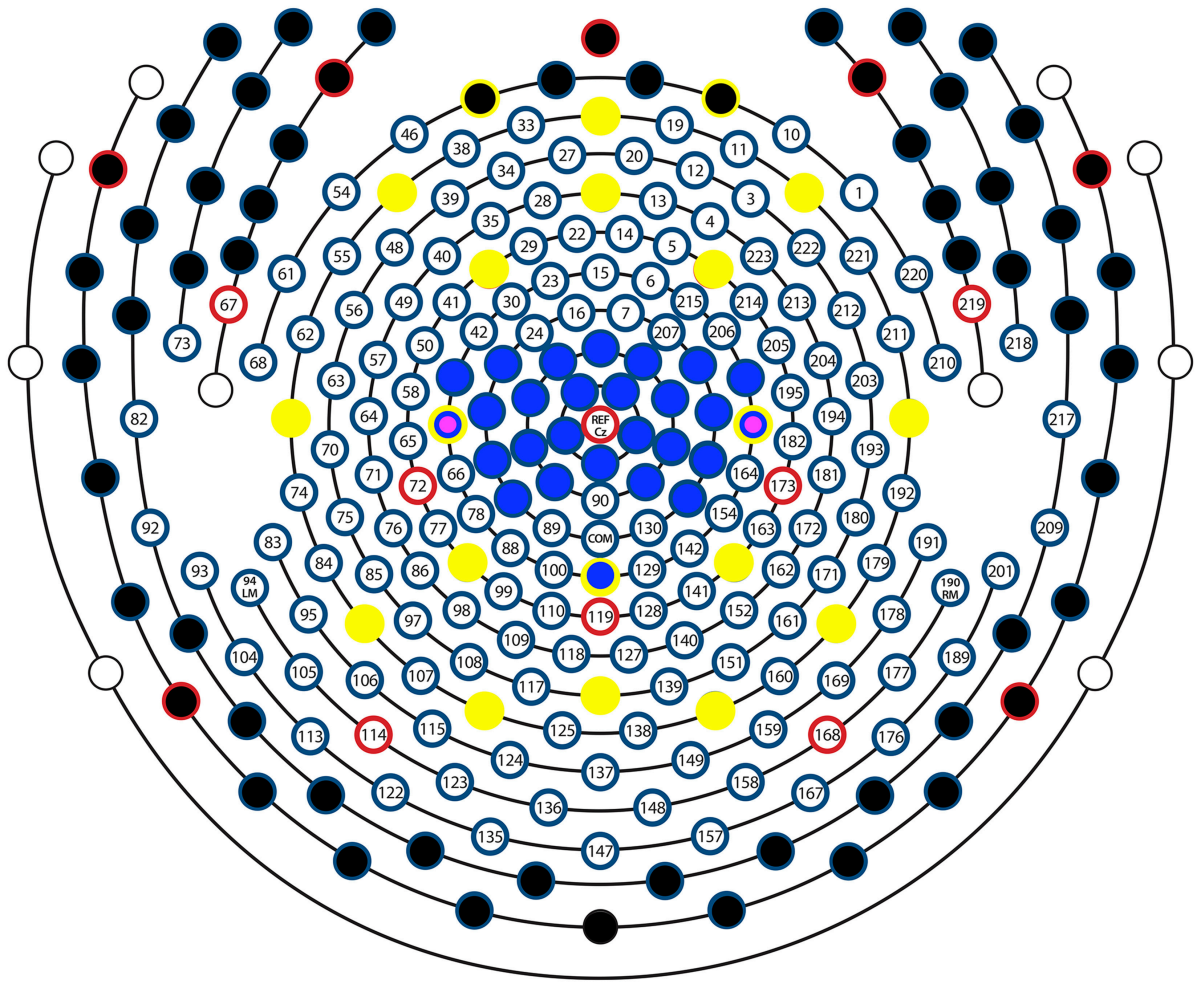
Sensor layout. Sensor layout of EGI's 256-channel sensor net, with the sensor configurations examined in this study marked with different colors. All channels except those with a black circle were used for the high-density whole brain configuration, all sensors with a blue circle were used for the high-density sensorimor configuration, all sensors with a yellow circle were used for the low-density whole brain configuration, and the two sensors with a magenta circle represent C3 and C4, used for the low-density sensorimotor configuration. Multiple overlaying colors represent channels that were used/not used in multiple configurations. E.g., Black and yellow indicates that this sensor was used in the low-density whole brain but not in the high-density whole brain configuration, while a yellow-blue-magenta circle means that the sensor was used in all 4 configurations. Red circles are retained from the original image, which is provided by EGI according to the manual. These electrodes serve as orientation points when mounting the nets. The electrode Cz was actually not used as an additional channel, but the signal from this location was included in all channels because it served as a reference.

Thus, for each segment, we formed 8 feature vectors to be submitted for classification analysis; 2 for the two measures ffDTF and FFT, and 4 for the 4 spatial configurations: low-density sensorimotor (central electrodes left and right in 10-10 electrode system, C3 and C4), low-density whole brain (all 19 10-10 electrode positions), high-density sensorimotor (27 channels from the central region of the HD-EEG sensor net), and high-density whole brain (all 256 channels of the HD-EEG sensor net except the above listed electrodes in the face and the neck, thus resulting in 197 channels). Figure 2 demonstrates the configurations.

The measure ffDTF was calculated with the functions mvfreqz.m and mvar.m from the BioSig toolbox (60). We chose model orders which satisfied the balance between the coefficients that needed to be estimated and available samples. That is, it was suggested that *N*/(*M*·*p*)>1 where N is the number of samples, M the number of channels, and *p* the model order. Given that we had up to 256 channels in the HD-EEG case and 250 samples for each of the 1-s segments, we had to exclude electrodes a priori in order to obtained a ratio of *N*/(*M*·*p*)>1 for a model order *p*. Therefore, we excluded the electrodes in the neck and in the face, since the electrodes in the face contain mostly muscle artifacts and those in the neck in addition low-quality data because of poor contact with the skin when participants had long hair.

Model orders were chosen for each of the 4 spatial configurations for the estimation of ffDTF, depending on the number of channels. We chose the maximum possible model order, which was *p* = 1 for the high-density whole brain configuration, *p* = 9 for the high-density sensorimotor configuration, *p* = 12 for the low-density whole brain configuration. However, we used *p* = 50 for the low-density sensorimotor configuration, since this is already a high model order ensuring high reliability (57). That way we follow the rule that the maximum possible model order should be chosen, and keep the ratio *N*/(*M*·*p*) consistent across conditions, except for the low-density sensorimotor configuration.

In order to increase robustness of the measures (57) we averaged the obtained values of FFT and ffDTF in frequency bands 1–2 Hz, then in 2 Hz bands up to 20 Hz, and then for 20–30, 30–40, and 40–48 Hz, thus, resulting in 13 frequency bands.

### 2.8. Classification analysis

We performed classification for pairs of conditions, separately for each type of spatial configuration, and for each recording of each participant. We compared each of the imagination and movement conditions with rest, and we compared the imagination of hand vs. foot, the movement of hand vs. foot, and the observation of hand vs. foot. We did not include a comparison of observation of hand or foot with rest, since the classification of observation of hand vs. foot should just demonstrate that the classification performance was mainly triggered by visual imagination.

In order to determine practicability of the different HD-EEG configurations we implemented a machine-learning approach which is likely to serve the system-training stage of a BCI, that is, supervised learning.

We decided to use support vector machines for classification, because they are well suited for non-linear properties of the data even when a linear kernel is used. When data are only non-linearly separable, the data is mapped into a feature space in which the linearly separating hyperplane can be used. We performed a classification in the sense of supervised learning with a linear kernel function (dot product) and quadratic programming in order to find the separating hyperplane, resulting in a 2-norm soft-margin support vector machine, by using the MATLAB^Ⓡ^ functions svmtrain and svmclassify from the statistics and machine learning toolbox.

### 2.9. Feature subset selection

**Figure 3:**
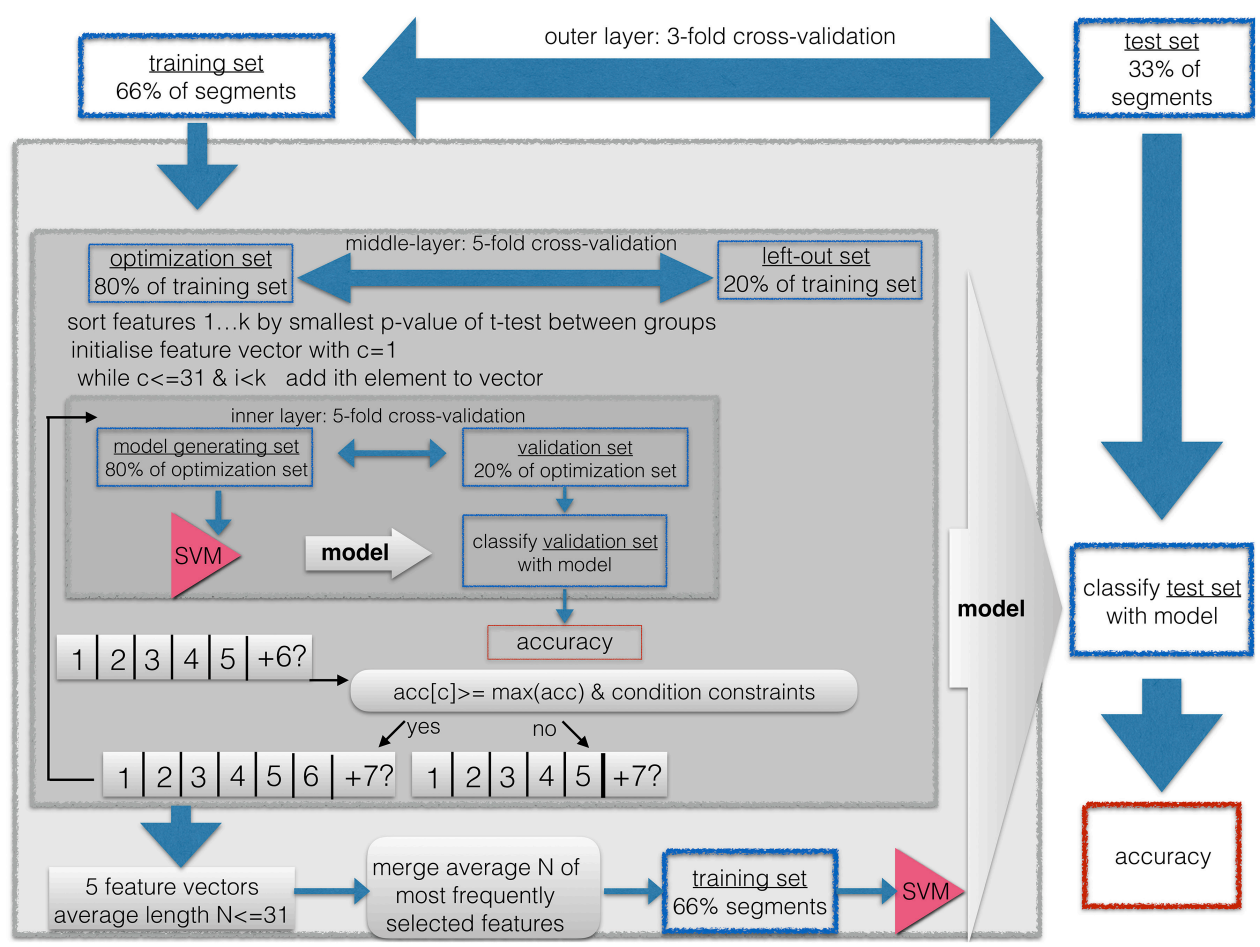
Classification and feature subset selection procedure. A nested-cross-validation procedure with an outer-loop for estimation of generalization and an inner-loop for feature vector optimization was implemented.

We performed a nested cross-validation with 3 layers with feature vector optimization, that is, feature subset selection as illustrated in Figure 3.

This procedure was used for three purposes. First, it is known that when the length of the feature vector exceeds the size of the sample, it can cause artificially high accuracies due to overfitting. Thus, shortening the feature vector to a length that is smaller than the training sample prevents us from running into the small sample size problem. This is easily the case for the ffDTF feature vectors, because then the length of the feature vector is up to *N*−*channels*×*N*−*channels*×*N*−*frequencies* = 197 × 197 × 13. Second, a long feature vector with uninformative features prevents the machine learning algorithm from finding a good solution. Therefore, the shortest possible feature vector should be found in the sense of a feature vector optimization. Third, we wanted to restrict the length of the feature vector to the shortest available number of features across the 4 spatial configurations and the two types of features (ffDTF and FFT). Because the maximally available features was 30 for the low density sensorimotor configuration and the FFT feature vector, we limited the maximally acceptable length of the feature vector to 30 entries in the inner loop of the feature vector optimization process, and 31 entries as a final selection.

As described in Figure 3, the classification and feature subset selection procedure was done in a nested design with 3 layers. Before submitting the segments to the classification, they were shuffled, so that the divisions into outer, middle, and inner layer contained a random set of segments from each of the conditions.However, we kept the subsegments of each trial (i.e., 6 subsegments per trial) within one subdivision, so that trials were strictly separated.

We implemented an outer layer as a 3-fold cross validation, and thus, a division of the data into one third of the data for testing the resulting model, and two thirds for feature vector optimization and cross validation, i.e., submitted to the middle layer. The middle layer is a first inner loop, implemented with 5-fold cross-validation. This loop aims to estimate the consistency of selected features, since each run yields a different feature vector. Thus, for each of the selected features one can count the times it was selected across the 5 runs. The inner layer is a second, thus, nested inner loop, again with 5-fold cross-validation in order to perform adequate feature subset selection. So-called k-fold cross-validation consist of *k* repetitions of leaving out *N*/*k* segments as the training set, while the remaining *N*−(*N*/*k*) segments are used during the training step.

All subsets were drawn in order to maintain the original proportion of the two groups.

Thus, the whole algorithm can be described as follows:

1. First, one third of the segments were excluded as the outer-layer test set for the final validation step in the outer layer, while the remaining two thirds of segments were used as the outer-layer training set, submitted to the next step
2. The outer-layer training set obtained from the outer loop was divided into 5 equal sized subsets, each one maintaining the proportion of condition sizes (25:25) from the original sample. For each of these 5 sets, the following steps were repeated:
  - The set was left out, the other 4 sets were merged to form the middle-layer training set.
  - A *t*-test for the middle-layer training-set segments was calculated between the two conditions, thus yielding one *p*-value for each entry of the feature vector.
  - The resulting *p*-values were sorted in ascending order.
  - The feature vector was initiated by taking the feature with the smallest *p*-value, thus, the initial length was one.
  - For this feature vector, the classification accuracy was calculated with 5-fold cross-validation, thus, the middle-layer training set was divided into an inner-layer 5-fold partition with an inner-layer training- and testing set
  - Now, the next feature from the sorted list was added. For this feature vector, the inner-layer classification with 5-fold cross-validation was repeated.
  - Now the result was compared to the previous result. The new entry to the feature vector was included only if the condition constraints were met as follows:
    - the classification accuracy obtained with the current feature vector was larger than or equal to the maximum of the previously obtained classification accuracies; that is, the second accuracy had to be ≤ than the first entry, or the 6th accuracy had to be ≤ than each of the five previous classification accuracies.
    - If the so far best sensitivity/specificity, or in other words, accuracy for segments of the first condition/second condition, respectively, was lower than 0.75, then the obtained sensitivity had to be ≤ than this maximum.
    - If the so far best specificity/sensitivity, was lower than 0.5, then the obtained specificity had to be larger, that is > than this maximum.
  - This way, features were added and tested for their contribution to the classification accuracy until all available features were used, or until the feature vector reached a maximum of 30 entries, or if more than a consecutive number of 10% of all available features was not added to the feature vector. If 10% was less than 100 features, than the maximum number of features that were tested was 100.
3. The average length *N* of the resulting 5 optimized feature sets was calculated. The number of times each feature was selected across these 5 runs was counted. A final feature vector was formed by including only those features which were selected at least in 2 of the 5 iterations. If this resulted in no features, all features were included that were selected at least in 1 out of 5 iterations. If the resulting feature vector included more than *N* features, only the top-most selected 31 features were included.
4. The resulting feature vector was used to train a support vector machine on the outer-layer training set, and the resulting model was used to classify the outer-layer test set, which was then used to calculate the general classification accuracy and the within-group accuracy for the two conditions (i.e., sensitivity/specificity).

The threshold of 0.75 was selected as rough estimators for above-chance classification. However, please note that this threshold is arbitrary, because it is not meant for interpretation of the results but for selection of features.

Feature subset selection and classification was done for each of the two feature types ffDTF and FFT, for each EEG recording, for each comparison of conditions, and for each spatial configuration, resulting in a total of 1008 classification accuracies.

### 2.10. Above-chance classification

As a result of classification, we got a classification accuracy for each feature and each combination of conditions. We considered whether the obtained accuracies were significantly above chance with the maximum chance criteria as described in Marcoulides and Hershberger (61). The maximum chance criterion *HC*_*max*_ is simply the maximum of the two sample sizes, which is equal in our case (150). We required that the accuracy should be significantly better than the chance criterion (61). That is, an accuracy is considered as reflecting task performance if the improvement over chance criterion was significant as assessed by a z-statistic:.

(1) $$\begin{array}{r} z=\frac{H_{0}-HC}{\sqrt{HC\left(N-HC\right)/N}} \end{array}$$

where *H*_0_ is the number of correctly classified trials and *N* is the total number of trials in both categories. The significance of z is determined according to the critical values from a standard normal distribution. This was done by computing the probability density function of the normal distribution with mean 0 and standard deviation 1 at the resulting *z*-values. Considering an alpha level of 0.05 this would result in a chance level of *H*_0_ = 167 = 55.66%. However, as we need to correct for multiple comparisons, the critical alpha level of 0.05 should be divided by 7*2 = 14 for the 7 conditions and 2 features. This resulted in a critical alpha level of *p* ≤ 0.0036 and a critical chance level of *H*_0_ = 175 = 58.43%.

If we consider that the chance level is not only related to the imbalance of the classes but to the number of trials in the test set, with an imbalance of 270:30, this would result in a critical chance level of *H*_0_ = 175 = 94.65%, thus, the chance level increases with the number of folds.

Since 58.43% cannot be considered an important classification result, we chose to use an arbitrary classification accuracy as a cut-off, i.e., 75%, which is more common in the classification accuracy than 58.43%.

### 2.11. Statistical analysis

We considered the between-subjects factor group (controls vs. patients), as well as the within-subjects-factors feature (ffDTF vs. FFT), comparison pairs (seven pairs as described in section 2.8), and spatial configuration (four configurations as described in section 2). These four factors were completely crossed. Therefore, it was technically possible to perform inference for all main effects and interactions. However, standard ANOVA, MANOVA, or repeated measures analysis methods could not be applied, due to the clear non-normal data distributions (classification proportions only take a discrete set of values between 0 and 1) and heterogeneous variances. The variance in the patient group was 0.0224, while for the healthy control group it was 0.0206.

Therefore, we chose a semi-parametric repeated measures ANOVA-type statistic that only requires metric data, but allows for non-normality and variance heterogeneity (62). This method is implemented in the R-package MANOVA.RM (63). We used the RM() function with the parametric bootstrap which showed the most favorable performance in unbalanced designs and was therefore, generally recommended (62).

The overall accuracy of a classification can be artificially high when one class is being perfectly classified while the classification accuracy is at chance in the other class. Therefore we assessed also the within-class accuracy and represented the distributions as boxplots for each group, type, and comparison.

The feature subset selection procedure could result in different lengths of the feature vector. Therefore, we tested whether the length of the feature vector was different between groups, features, comparisons, and spatial configurations. The number of features is ordinal, therefore again, we used the non-parametric ANOVA-type statistic as for the comparison of classification accuracies.

Since we performed analysis for each recording of each participant we had multiple recordings for most patients, but for none of the healthy controls. We needed to rule out that the patients had an advantage on the subsequent recordings because of learning effects. Therefore, we performed the ANOVA-type statistic again by including only the first sessions of the patients. We did not restrict the analysis to these first recordings in general, because then the two sample sizes are even more different, and the generalizability of the effect can be characterized better by interpreting the results of both statistical analysis, the one for the first recordings only, and the one including all recordings.

## 3. Results

### 3.1. Sample

Among the 22 healthy controls were 14 women, and the age ranged from 19 to 35 years (mean = 23.14, *SD* = 3.4). We recruited seven male patients with traumatic spinal cord injury at the Department of Neurology. The patients were in an age range between 24 and 70 years (mean = 51.86, *SD* = 5.49). All patients were right-handed. Details on the level and extent of injury, number of recordings as well as time since injury are given in Table 1.

**Table 1:** Clinical details of the patients with SCI included in the study and number of EEG recordings of each patient.

**Code**	**Age**	**Sex**	**Etiology**	**AIS**	**Injury**	**Time**	**n**.	**Hand**	**Foot**
1	51	m	Cervical myopathy;	D	C4-C7	2	2	None	++motor
			laminectomy						+ sensory
2	61	m	Resection of intramedullar	C	C5-C6	204	4	+strength	No movement, spasms
			meningeoma WHO grade II					+fine motor	++sensory
3	24	m	Fracture	C	C5-C6	48	2	+motor	++motor
								spasms	Pain sensation
4	65	m	Fracture; luxation	C	C3-C4	19	3	++motor	No movement
								++sensory	++sensory
5	44	m	Fracture	D	C5-C6	216	1	++motor	No movement
								++sensory	No sensation
6	48	m	Fracture	D	C6-C7	312	1	+motor	No movement
								+sensory	No sensation
7	70	m	Spinal ischemia T8	D	T4-T8	12	1	None	+motor
			arteriovenous fistula T4-8						+sensory
			laminectomy L4/5

*AIS, ASIA scale; time, in months; injury, location; n., number of recordings; hand/foot, disabilities; ++, strong; +, mild*.

### 3.2. Classification accuracies

Table 2 shows the average accuracies across participants, separately for each group.

**Table 2:** Average accuracies in % separately for each group, space (LD-SM, C1 and C2 channels; LD-whole, low density full montage; HD-SM, high density sensorimotor cortex; HD-whole, high density full montage), comparison (7 pairwise comparisons of conditions), and feature (ffDTF vs. FFT).

	**Controls**				**Patients**			
**Comparison**	**LD-SM**	**LD-whole**	**HD-SM**	**HD-whole**	**LD-SM**	**LD-whole**	**HD-SM**	**HD-whole**
**FFT**								
IF-RS	55	57	57	56	61	62	60	62
IH-RS	54	56	56	56	63	63	60	63
MF-RS	64	74	75	78	68	69	70	73
MH-RS	63	66	65	68	66	70	68	70
IF-IH	49	49	51	50	51	51	50	47
MF-MH	59	71	72	74	54	61	57	62
OF-OH	50	49	51	50	51	51	48	46
**ffDTF**								
IF-RS	54	54	52	52	58	56	54	57
IH-RS	54	52	52	51	60	57	55	59
MF-RS	61	62	64	65	62	60	60	62
MH-RS	60	57	57	56	60	60	58	59
IF-IH	51	49	51	48	52	48	50	49
MF-MH	56	59	61	63	51	55	53	56
OF-OH	49	49	50	49	50	49	50	49

*LD, low density; SM, sensomotoric region; HD, high density; FFT, fast fourier transform; ffDTF, full frequency directed transfer function; IF, imagine foot movement; IH, imagine hand movement; RS, rest; MF, move foot; MH, move hand; OF, observe foot movement; OH, observe hand movement*.

Table 3 shows the number of healthy participants and the number of recordings/first recordings from patients with above >0.75 accuracy, separately for each group.

**Table 3:** Number of healthy participants and number of all recordings (14)/number of first recordings (7) from patients with above >0.75 accuracy, separately for each group, space (LD-SM: C1 and C2 channels; LD-whole, low density full montage; HD-SM, high density sensorimotor cortex; HD-whole, high density full montage); comparison (7 pairwise comparisons of conditions), and feature (ffDTF vs. FFT).

	**Controls**				**Patients**			
	**LD-SM**	**LD-whole**	**HD-SM**	**HD-whole**	**LD-SM**	**LD-whole**	**HD-SM**	**HD-whole**
**FFT**								
IF-RS	0	0	0	0	4/1	3/0	4/1	3/0
IH-RS	0	0	1	0	3/0	3/0	3/0	3/0
MF-RS	5	9	8	11	1/0	4/2	4/2	6/3
MH-RS	1	1	2	3	2/1	4/1	4/1	4/1
IF-IH	0	0	0	0	0/0	0/0	0/0	0/0
MF-MH	2	9	9	10	0/0	0/0	0/0	1/0
OF-OH	0	0	0	0	0/0	0/0	0/0	0/0
**ffDTF**								
IF-RS	0	0	0	0	1/0	0/0	0/0	0/0
IH-RS	0	0	0	0	0/0	0/0	0/0	0/0
MF-RS	0	3	3	4	1/1	0/0	0/0	0/0
MH-RS	1	0	0	0	0/0	0/0	0/0	0/0
IF-IH	0	0	0	0	0/0	0/0	0/0	0/0
MF-MH	0	3	4	4	0/0	0/0	0/0	0/0
OF-OH	0	0	0	0	0/0	0/0	0/0	0/0

*LD, low density; SM, sensomotoric region; HD, high density; FFT, fast fourier transform; ffDTF, full frequency directed transfer function; IF, imagine foot movement; IH, imagine hand movement; RS, rest; MF, move foot; MH, move hand; OF, observe foot movement; OH, observe hand movement*.

The accuracies and numbers of participants with above >0.75 classification are higher for the FFT than the ffDTF, and higher for the comparisons that involve movement conditions, especially the move-foot condition. Applying the strict chance level of >0.95, no above-chance classifications are obtained for ffDTF and no for the patients and FFT. Only the larger configurations than low density sensomotoric region obtained classification accuracies above this for move foot vs. rest (4 healthy controls for each of the three configurations) and for the move foot vs. move hand comparison (three for the low density full-montage configuration and 5 for the two HD-configurations).

Table 4 shows the statistical results of the repeated-measures ANOVA-type statistic. Due to repeated sessions in 4 patients, one could assume that these 7 follow-up sessions are biased by learning effects. Therefore, we performed the analysis again by excluding all follow-up sessions and interpreted all results on the Bonferroni-corrected level of significance, which is *p* < 0.025 because of the two test statistics.

**Table 4:** Statistical results for the non-parametric ANOVA-type statistic for repeated measures designs, examining the effects of group (controls vs. patients), space (C1 and C2 channels vs. low density full montage vs. high density sensorimotor cortex vs. high density full montage), comparison (7 pairwise classifications of conditions), and feature (ffDTF vs. FFT).

	**Whole sample**			**First recordings**		
**Factor/interaction**	**F**	**df1**	***p*-value**	**F**	**df**	***p*-value**
Group	0.17	1	0.68	4.01	1	0.04
Space	1.73	2.96	0.16	2.79	2.94	0.04
Comparison	100.36	4.92	<0.001	101.91	4.98	<0.001
Feature	371.78	1.00	<0.001	199.41	1	<0.001
Group:space	2.21	2.956	0.09	1.75	2.94	0.16
Group:comparison	13.94	4.92	<0.001	13	4.98	<0.001
Space:comparison	1.88	14.33	0.02	1.68	14.19	0.05
Group:feature	0.16	1	0.69	2.46	1	0.12
Space:feature	12.50	2.97	<0.001	6.4	2.89	<0.001
Comparison:feature	36.81	5.74	<0.001	14.8	5.73	<0.001
Group:space:comparison	0.38	14.33	0.98	0.50	14.19	0.93
Group:space:feature	2.95	2.97	0.03	2.54	2.89	0.06
Group:comparison:feature	2.73	5.74	0.01	2.39	5.73	0.03
Space:comparison:feature	1.12	16.52	0.33	1.28	15.36	0.20
Group:space:comparison:feature	0.45	16.52	0.97	0.62	15.36	0.86

*The analysis was performed once for the whole sample of EEG data (whole sample), and once with only one EEG file per participant (first recordings)*.

*F, ANOVA type statistics; df, degrees of freedom*.

According to Table 4, the main effects of group and space were stronger in the analysis of the first recordings, but not for the whole sample, although after correcting for performing the ANOVA twice, the effect was not significant at the Bonferroni-corrected level of α < 0.025. The interactions between space and comparison and between group, comparison and feature were significant only based on the whole sample of EEGs, and not when the sample was restricted to the first EEG-recordings. However, when looking at the figures, we found that these differences were merely an effect of the size of variance of the data and not of the direction of the effect. Therefore, we represent figures of the whole sample, including repeated sessions.

**Figure 4:**
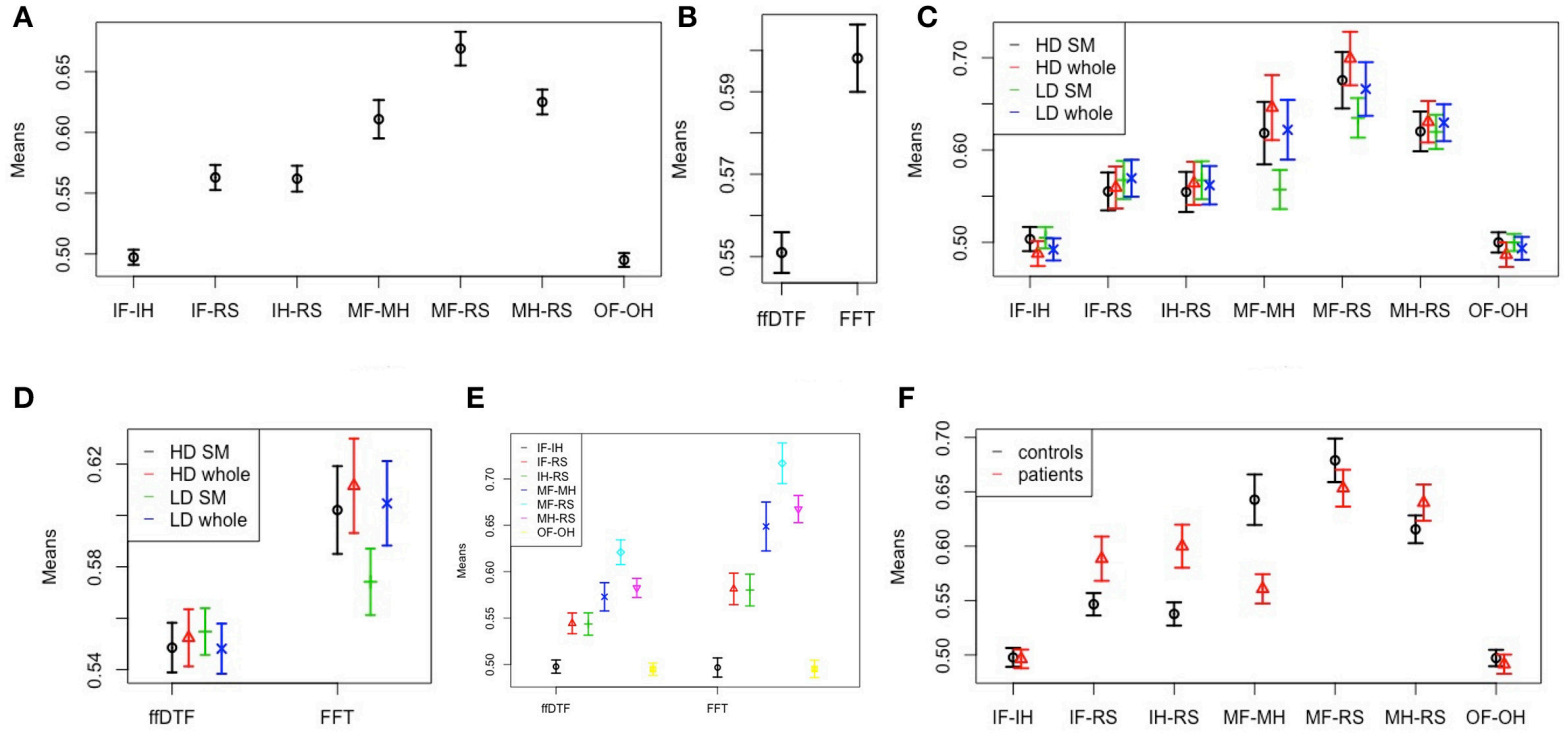
Effects and interactions. Means and 95% confidence intervals of single factors and interactions based on the non-parametric ANOVA-type statistic on classification accuracies. **(A)** Main effect comparison; **(B)** main effect feature; **(C)** interaction space:comparison; **(D)** interaction space:feature; **(E)** interaction feature:comparison; **(F)** interaction group:comparison. HD, high density; LD, low density; SM, sensomotoric cortex montage; whole, whole brain montage; FFT, Fast Fourier Transform; ffDTF, full frequency directed transfer function; IF, imagine foot movement; IH, imagine hand movement; RS, rest; MF, move foot; MH, move hand; OF, observe foot movement; OH, observe hand movement.

Figure 4 illustrates the main effects and comparisons. We found a main effect for comparison (Figure 4A). The movement conditions were better distinguishable from the other conditions and from each other than any other comparison. This is followed by the classification of imagining foot or hand movement vs. rest. The classification of the two imagery conditions against each other and the classification of the two observation conditions against each other yielded classification accuracies at chance level.

According to the main effect for measure (Figure 4B), FFT lead to substantially higher classification accuracies than ffDTF. Moreover, we found an interaction between comparison and space (Figure 4C). The differences between electrode configurations where greater for the configurations involving movement of the foot, and in general quite small for all other comparisons. The interaction between spatial configuration and measure (Figure 4D) indicated that for ffDTF the differences between spatial configurations were negligible, while for the FFT there was a considerable difference. The worst result was obtained by using only the sensomotoric electrodes from the 10–20 system, followed by the high-density montage of the sensomotoric cortex and low-density whole brain montage. Results based on high-density whole brain montage where slightly better, although statistically not distinguishable from the low-density whole brain montage and high density sensorymotoric regions.

The interaction between space and feature is illustrated in Figure 4E. In general, the accuracies are larger for FFT except for the comparison of imagination conditions to each other and observation conditions to each other, which are always at chance.

The interaction between group and comparison is highly significant (Figure 4F). The average classification accuracies were on average equal or higher in patients compared to controls in the comparison of imagination vs. rest, and in move hand vs. rest. In contrast, the average accuracies of the move foot vs. move hand and move foot vs. rest comparisons were higher in controls than in patients. For the classification of observation conditions against each other, and the imagination conditions against each other the confidence intervals of the two groups overlapped, and are in general at chance.

Finally, there was a three way interaction of group, comparison, and feature, which was significant only for the whole group but not when restricting the sample to the first recordings. The interaction indicated the larger differences for FFT than ffDTF. For ffDTF, the groups did not differ in the comparisons of imagine to move foot vs. imagine to move hand, while for FFT patients performed slightly better. Because of the three-way interaction we performed another analysis stratified by feature. The results for the separate ANOVA-type statistic for FFT and ffDTF are shown in Table 5.

**Table 5:** Statistical results for the non-parametric ANOVA-type statistic for repeated measures designs, separately for the two feature types FFT and ffDTF, examining the effects of group (controls vs. patients), space (C1 and C2 channels vs. low density full montage vs. high density sensorimotor cortex vs. high density full montage), and comparison (7 pairwise comparisons of conditions).

	**FFT**			**ffDTF**		
**Factor/interaction**	**F**	**df**	***p*-value**	**F**	**df**	***p*-value**
Group	0.011	1	0.917	0.25	1	0.62
Space	2.17	2.92	0.09	0.46	2.93	0.70
Comparison	125.51	3.45	<0.001	79.90	4.36	<0.001
Group:space	1.18	2.9	2.32	0.62	2.93	0.6
Group:comparison	15.16	3.45	<0.001	14.13	4.36	<0.001
Space:comparison	2.21	9.9	0.02	2.18	11.95	0.01
Group:space:comparison	0.46	9.9	0.92	0.57	11.95	0.87

*F, ANOVA type statistics; df, degrees of freedom*.

**Figure 5:**
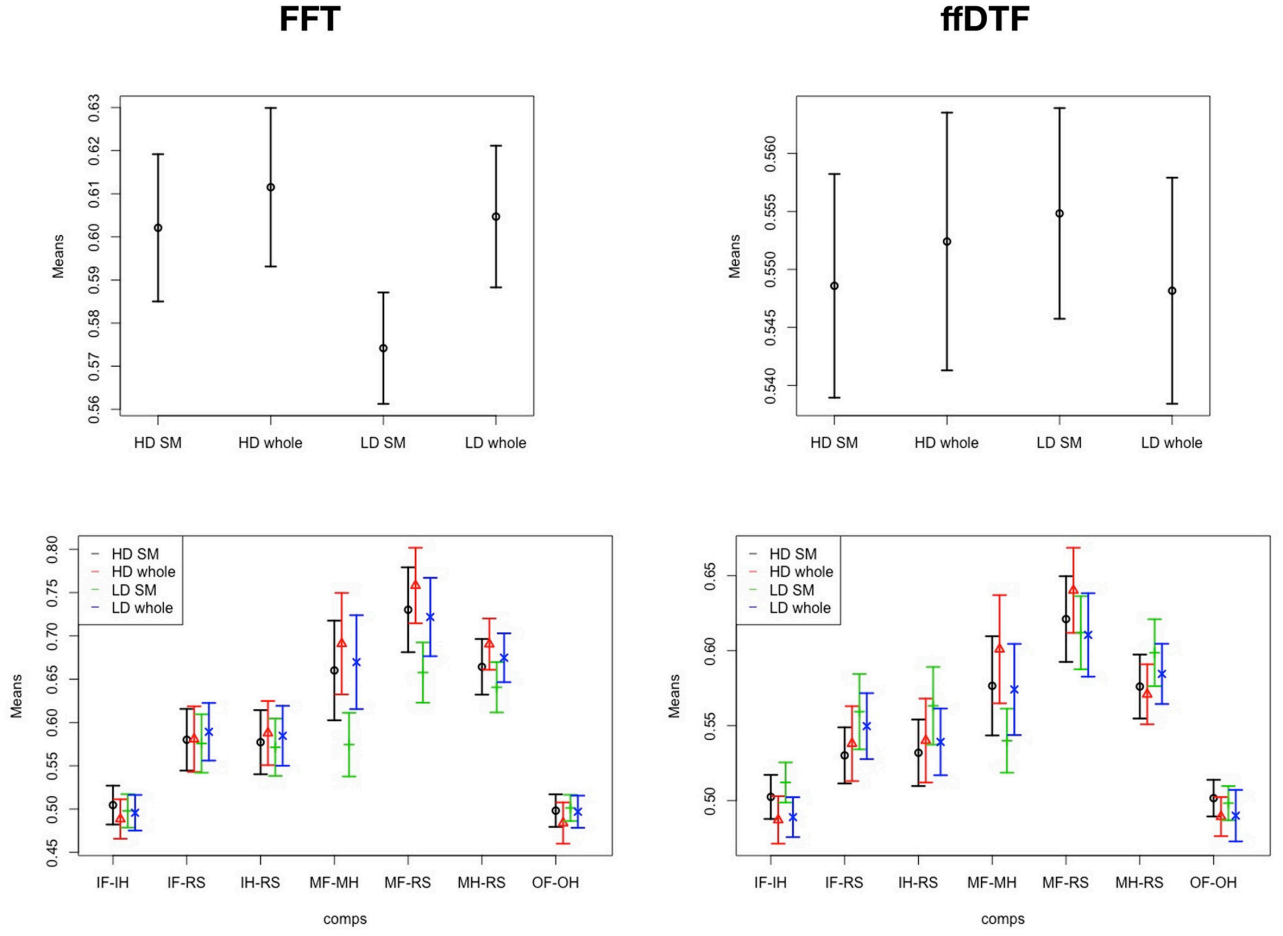
Effects and interactions. Means and 95% confidence intervals of significant main effect of spatial configuration **(Top row)** and interaction space:comparison **(Bottom row)** based on the non-parametric ANOVA-type statistic on classification accuracies, separately for FFT, Fast Fourier Transform; ffDTF, full frequency directed transfer function; HD, high density; LD, low density; SM, sensomotoric cortex montage; whole, whole brain montage; IF, imagine foot movement; IH, imagine hand movement; RS, rest; MF, move foot; MH, move hand; OF, observe foot movement; OH, observe hand movement.

The results of the two tests seem to be comparable. The ANOVA scores for space and comparison are larger for FFT than for ffDTF. Figure 5 indicates that the difference in the main effect space is due to the fact that the interaction in ffDTF levels out the differences for the main effect of space. The comparisons with movement of the foot show the same direction of the influence of space for both measures, while FFT shows no differences for the imagination conditions, whereas the effect seems to change a lot for ffDTF, however with in general very low values.

**Figure 6:**
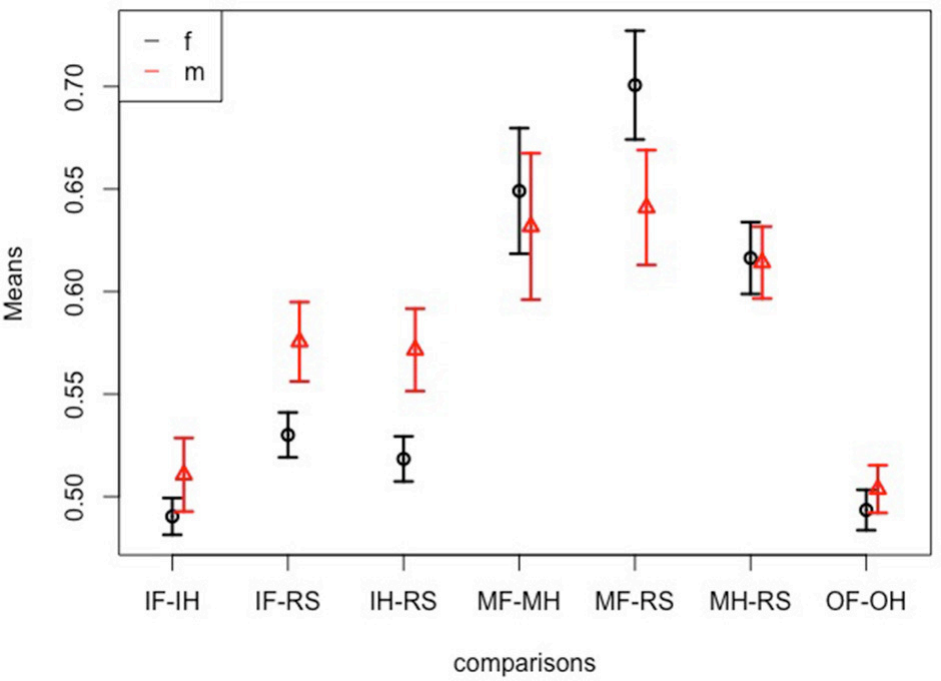
Sex effects. Means and 95% confidence intervals of significant interaction sex:comparison based on the non-parametric ANOVA-type statistic on classification accuracies; IF, imagine foot movement; IH, imagine hand movement; RS, rest; MF, move foot; MH, move hand; OF, observe foot movement; OH, observe hand movement.

Because the patient group included only men and the control group both sexes, we conducted a further ANOVA-type statistic by including only controls and by considering the factor sex instead of group. According to the ANOVA-type statistics there was a significant interaction of sex and comparisons [*F*_(3.8)_ = 4.62; *p* < 0.001]. Figure 6 shows that men showed better classification accuracies than women for the comparisons of imagined movement vs. rest, while women showed higher accuracies for movement of the foot vs. rest. There were no further interactions.

### 3.3. Classification characteristics

**Figure 7:**
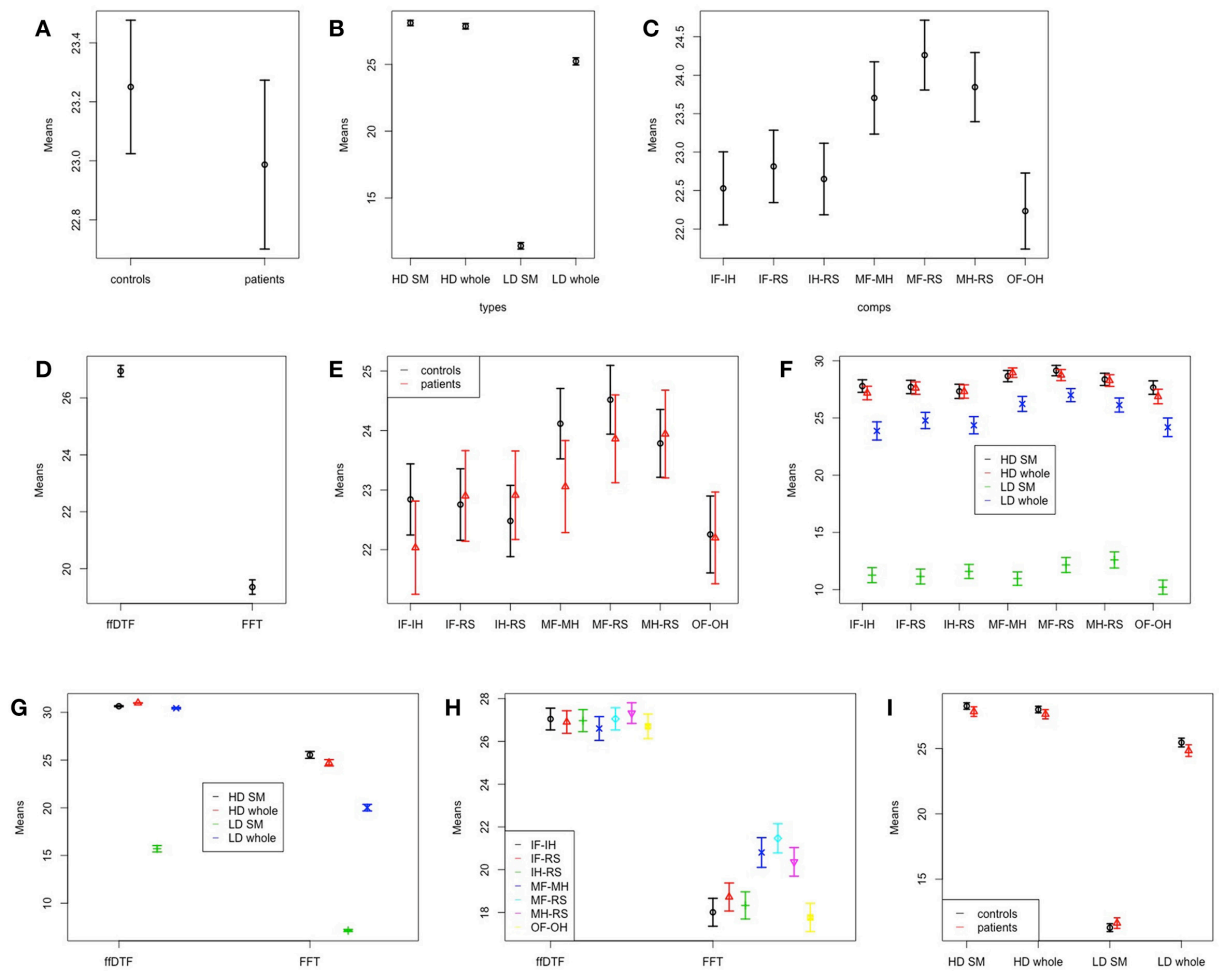
Effects and interactions for feature vector sizes. Means and 95% confidence intervals of significant single factors and interactions based on the non-parametric ANOVA-type statistic on feature vector sizes after feature subset selection. **(A)** Main effect group; **(B)** main effect space; **(C)** main effect comparison; **(D)** main effect feature; **(E)** interaction group:comparison; **(F)** interaction space:comparison; **(G)** interaction space:feature; **(H)** interaction comparison:feature; **(I)** interaction group:space. HD, high density; LD, low density; SM, sensomotoric cortex montage; whole, whole brain montage; FFT, Fast Fourier Transform; ffDTF, full frequency directed transfer function; IF, imagine foot movement; IH, imagine hand movement; RS, rest; MF, move foot; MH, move hand; OF, observe foot movement; OH, observe hand movement.

We tested for differences in feature vector length after feature subset selection (Table 6 and Figure 7). The feature vector length after feature subset selection is larger for controls than for patients (Figure 7A). Moreover, the feature vector length varies largely between spatial configuration (Figure 7B), between comparisons (Figure 7C), and between the two features (Figure 7D). The interaction between group and comparison (Figure 7E) is reflected by shorter feature vectors in the patient group for the comparison of move foot vs. move hand. The interaction between spatial configuration and comparison (Figure 7F) is not that clear, because the low-density sensorimotor configuration results in very short feature vectors compared to other configurations. The interaction between spatial configuration and feature (Figure 7G) reflects a smaller difference for ffDTF between all but the low-density sensorimotor configuration, while the differences are larger for FFT. The interaction between comparison and feature (Figure 7H) is such that the conditions differ between each other more strongly for FFT than for ffDTF. Finally, the interaction between group and spatial configuration (Figure 7I) is not very strong. There seems to be slightly shorter feature vectors for patients than controls for all comparisons except for the low-density sensimotoric configuration, where the direction seems to be the other way round.

**Table 6:** Statistical results for the non-parametric ANOVA-type statistic for repeated measures designs, examining the sizes of feature vectors after feature subset selection with effects of group (controls vs. patients), space (C1 and C2 channels vs. low density full montage vs. high density sensorimotor cortex vs. high density full montage), comparison (7 pairwise comparisons of conditions), and feature (ffDTF vs. FFT).

**Factor/interaction**	**F**	**df**	***p*-value**
Group	8.39	1	0.004
Space	7504.38	2.972	<0.001
Comparison	39.53	5.97	<0.001
Feature	7770.35	1	<0.001
Group:space	5.47	2.97	001
Group:comparison	5.607	5.97	<0.001
Space:comparison	3.873	17.56	<0.001
Group:feature	37.23	1	<0.001
Space:feature	186.35	2.98	<0.001
Comparison:feature	39.1	5.99	<0.001
Group:space:comparison	3.22	17.56	0.001
Group:space:feature	0.76	2.98	0.52
Group:comparison:feature	11.59	5.99	<0.001
Space:comparison:feature	4.51	17.74	<0.001
Group:space:comparison:feature	2.03	17.74	0.006

*F, ANOVA type statistics; df, degrees of freedom*.

In general, the classification accuracy seems to be better with a longer feature vector, except for the fact that the classification based on FFT results in a lower feature vector length, but also in higher classification accuracies compared to ffDTF.

**Figure 8:**
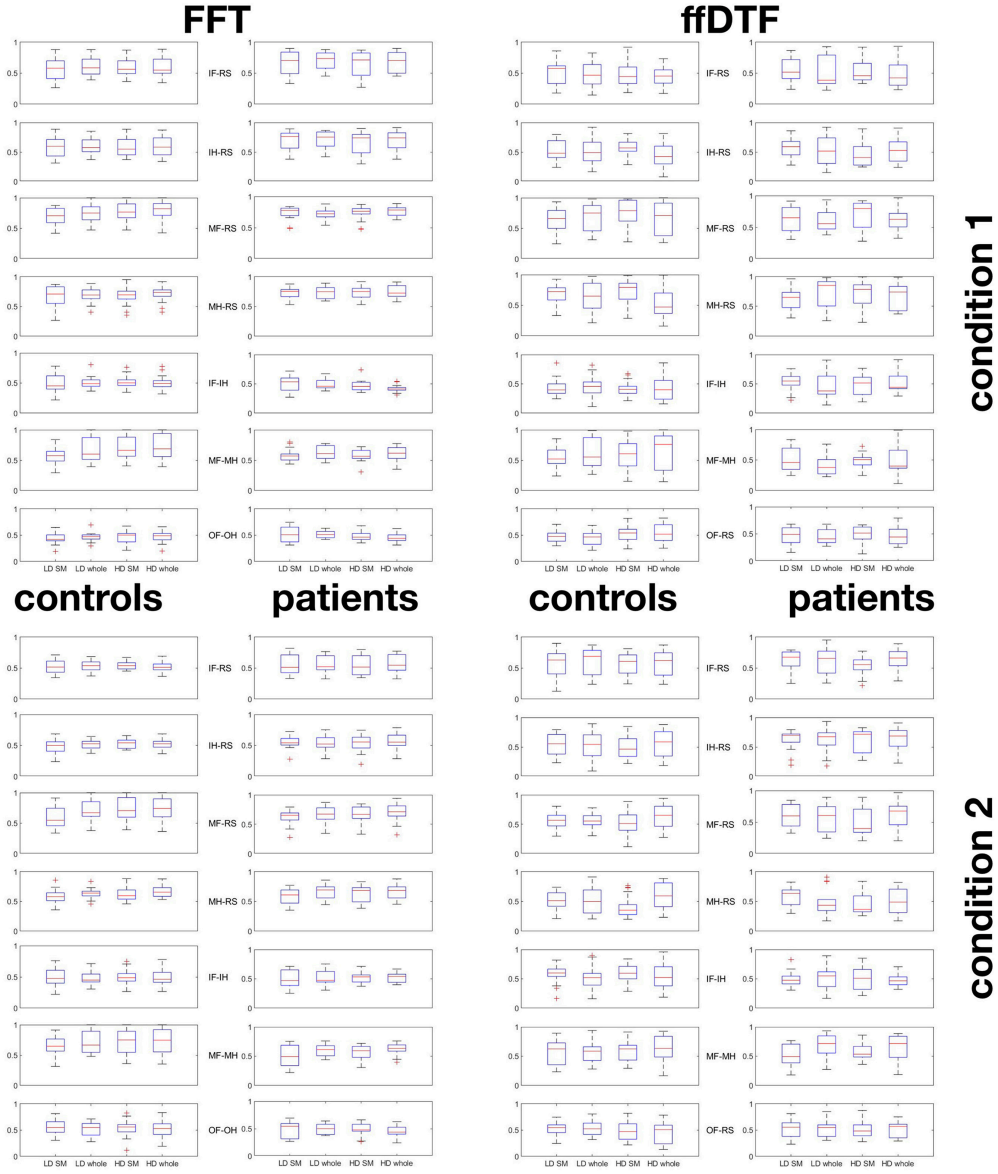
Within-condition accuracies. Boxplots indicating that pairwise classifications were not due to overfitting to one of the two conditions. First block represents condition 1, second block condition 2: For each pair-wise comparison of conditions, classification accuracies for the first condition is give in the top 7 plots, and for the second condition in the bottom 7 plots. Plots are given separately for groups of controls (first and third column) and patients (second and fourth column), for FFT (first and second column) and ffDTF (third and fourth column). The boxplots represent the range between the first and the third quartile as a box alongside with the median (red line in the middle of the box), and the whiskers are drawn to ±2.7σ, that is 99.3% coverage and extended to the adjacent value, which is the most extreme data value that is not an outlier. Outliers are represented as red crosses and defined as values that are greater than *q*_3_+1.5·(*q*_3_−*q*_1_) where *q*_*i*_ is the *ith* quartile. HD, high density; LD, low density; SM, sensomotoric cortex montage; whole, whole brain montage; FFT, Fast Fourier Transform; ffDTF, full frequency directed transfer function; IF, imagine foot movement; IH, imagine hand movement; RS, rest; MF, move foot; MH, move hand; OF, observe foot movement; OH, observe hand movement.

In order to demonstrate that the classification accuracies were not a result of overfitting the data to one of the two classes, we plotted the within-class accuracies in Figure 8. These accuracies can be interpreted in terms of sensitivity or specificity toward the one or the other condition. As there is no clear trend of single conditions to yield very low (i.e., below chance) accuracy while the other condition yields very high accuracy, our optimization method did not converge at the cost of single conditions.

**Figure 9:**
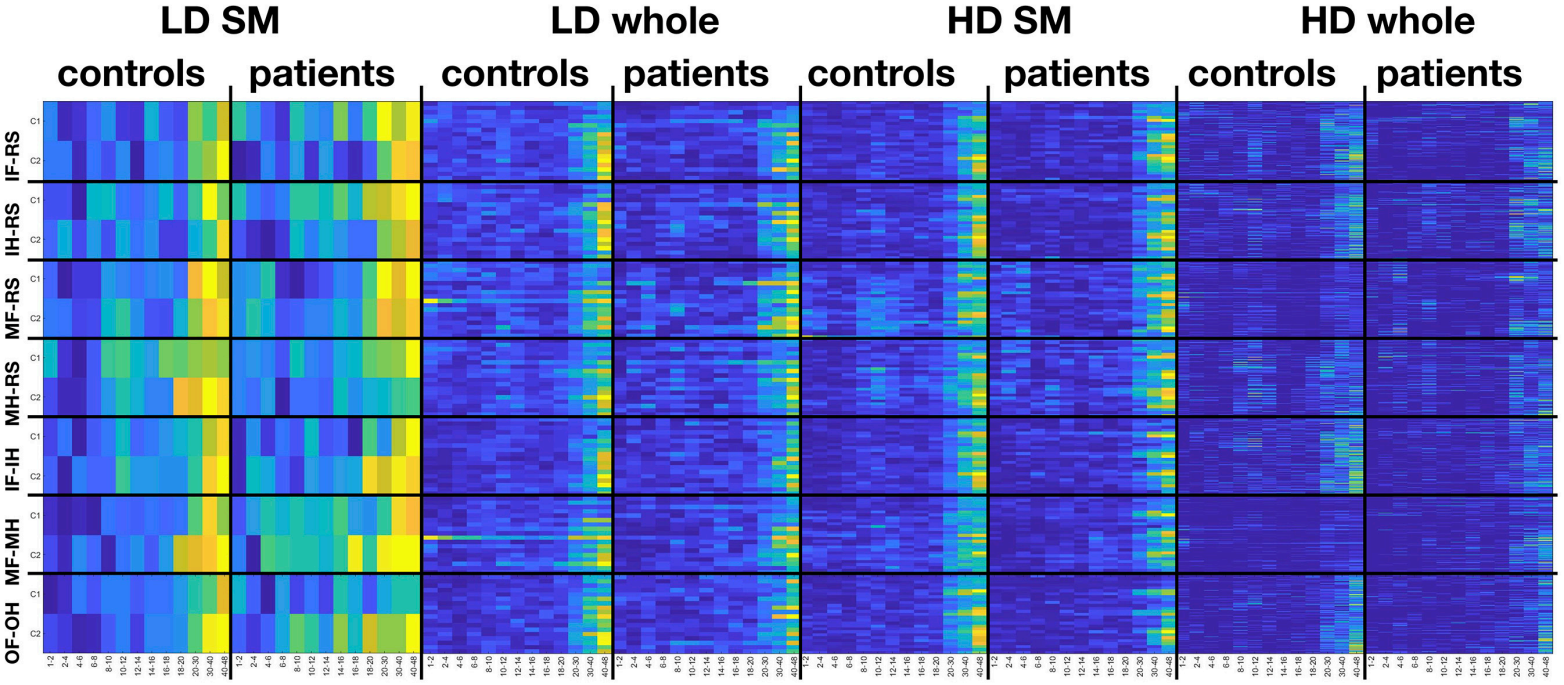
Frequency-electrode heatmaps. Heatmaps for the selected features from the FFT feature vector the four configurations in space, separately for controls and patients and pair-wise classification of conditions. Colors indicate percent from zero (dark blue) to 100 (yellow), indicating the proportion of participants of the respective sample for which the respective feature was selected. HD, high density; LD, low density; SM, sensomotoric cortex montage; whole, whole brain montage; FFT, Fast Fourier Transform; ffDTF, full frequency directed transfer function; IF, imagine foot movement; IH, imagine hand movement; RS, rest; MF, move foot; MH, move hand; OF, observe foot movement; OH, observe hand movement.

Figure 9 indicates which features were selected for the FFT. In the low density configuration with only two channels C1 and C2 there was a pronounced usage of higher frequencies

in the beta and especially gamma range. Also for the other three configurations there was a pronounced use of higher frequencies. However, the high density configurations, especially the sensorimotor configuration, are also characterized by a marked use of the μ rhythm around 8–12 Hz.

## 4. Discussion

We hypothesized that low-density EEG with coverage of the central regions, that is, the motor cortex, is sufficient to achieve good BCI performance in healthy participants and that the other regions do not contribute additionally to the performance while in patients with SCI the use of whole-head recordings as well as dense sampling might be more informative. In contrast to our hypothesis, we found that patients showed classification accuracies which were at least as good as those of healthy controls for the imagination conditions. Only for the conditions which involved movement of the foot, the classification accuracies obtained by the patients' signals were lower than the control group, which is most likely the case because often, they were actually not able to move the foot. Moreover, most configurations of electrodes lead to similar results, except for a poor classification accuracy for low-density sensorymotor coverage. Specifically, groups did not benefit differentially from the variation of montages. Finally, classification accuracies were higher with FFT than with ffDTF.

This result is somewhat surprising, since previous studies reported low accuracies when classifying motor imagery in patients (29). We think that some technical details may allow to explain this higher classification accuracies in the patient group of our study.

### 4.1. Frequency is more informative than synchrony

We found that by use of the FFT feature vector, higher classification accuracies could be obtained than with ffDTF. Indeed, examination of power modulations of specific frequency bands in the EEG have a long tradition in movement research, but also in SCI research.

Following a traumatic event with SCI, the spinal cord and the cortex become atrophic (64, 65) and cortical reorganization takes place (66). This is well known to be detectable also in the EEG as an immediate slowing of cortical activity after SCI (67) and a shift of motor potentials to a more posterior location, which is strongly related to recovery (68, 69). Boord et al. (70) and Tran et al. (71) described similar EEG findings in humans with SCI, as they compared patients with paraplegia, with or without neuropathic pain, and able-bodied controls. The reported slowing of cortical activity after SCI could be a correlate of the process which establishes cortical reorganization after SCI (72). We could possibly observe a similar effect in our data. In Figure 9 controls show selections of features from the 8–12 Hz bandwidths for the classification of hand movement vs. rest based on HD-EEG configurations, while these selections are less prominent and possibly shifted to a lower frequency range of 6–10 Hz in patients.

We suggest that in parallel to the cortical slowing related to SCI, also the activation related to motor imagery and movement observation changes. It seems that the frequency information, even if drifted to a lower frequency range, is still more informative to distinguish movement-related conditions than interactions between signals on the scalp. The most classical approach of motor-related biomarkers in the EEG are modulations of power in specific frequency bands. In most subjects, over the sensorimotor cortex activity in a frequency range between 8 and 30 Hz responds to real movement and to motor imagery (73–76), which is also reflected by our results. This range includes the mu (μ, 8–13 Hz) and beta (β, 14–30 Hz) oscillations. Activity in the μ-range is the most common starting point for detection of motor-related thoughts. Pineda (74) suggest that the μ-rhythm reflects the translation of visual or auditory cues into actions.

Unfortunately, there is considerable variance in motor imagery related activation. As summarized, typically, we would expect a desynchronization of the μ- and/or β-rhythm during movement or motor imagery. However, in some subjects and under certain circumstances, the μ- and/or β-rhythm synchronizes during imagination of movements, for example in single subjects during hand imagery (77), or during imagination of foot- and tongue-movement (75). It was suggested that patients with SCI show altered patterns of adjustment to mental fatigue in the μ-range (78). In contrast, power changes in the μ-range could already be found before an action is taken, and pre-action activation was also successfully used for control of a BCI (79). The inclusion of movement planning related activity from broader activation areas was shown to be extremely useful in patients with SCI (80). Indeed, in contrast to the focal event related power modulations in the β-frequency range of healthy subjects, patients with paraplegia exhibit diffuse power modulations during attempted foot movements (81). In this sense, the inclusion of EEG signals from the whole scalp seems to address this diffuse activation adequately. Spatial effects of neuroplasticity may affect the EEG as such that the fine-grained spatial coverage of HD-EEG delivers further information. However, the advantage of HD-EEG over LD-EEG in the full montage configurations was negligible. It is possible that the advantage of a better spatial coverage of the HD-EEG was leveled out by the complexity of the feature vector, which increased the demands on proper feature-subset selection.

Still, this does not fully explain why our patient data resulted in classification accuracies that were at least as good as those obtained from healthy controls. The effect was present more strongly as an interaction, while the main effect of group did not reach significance when using all recordings after correction for multiple comparisons. Since the group effect was stronger when restricting the analysis to the first recordings of the patient group, we can assume that the advantage of the patients is not due to learning effects over multiple sessions. In addition, we could speculate that the change of activity in other frequencies is easier to be detected than when restricting the analysis to the μ- and β-range. For example, the amplitude changes in lower frequencies are always at a larger scale than the higher-frequency changes. Therefore, we recommend to include a wider range of frequencies to the design of BCIs.

### 4.2. Control signals

In patients with SCI the ability to perform motor imagery, the mental activity which is mostly used to control BCIs, is impaired (82). Therefore, modulations of the μ- or any other rhythm cannot be taken for granted in this patient population. As an alternative, non-motor imagery could be used (83), or observation of movements and visual instead of kinesthetic imagination techniques could be implemented (84). It was found that patients have less difficulties in visual imagination than kinesthetic imagination (85). As we did not prescribe the strategy in this study, it is possible that patients implemented this type of imagination as a coping strategy. This could have been triggered by the observation conditions. It is possible that the participants tried to mentally re-play the observed movement during the imagination conditions. However, at least with respect to the selected features, the resemblance between the pairwise classification of the observation and imagination conditions is not overwhelming.

Most interestingly, the information contained in the EEG of patients led to slightly better classification accuracies than in controls for the imagery conditions, which clearly contradicts earlier findings, where the performance was better in healthy controls than in patients with SCI (29). However, the samples of our study and of Müller-Putz are not comparable, since their patients suffered from complete injury, while the patients in our study were diagnosed with AIS scores of C or D. A quite plausible explanation could be that there was an interaction between sex and condition in the control group. Men showed higher classification accuracies than women in the imagery conditions, while women showed higher accuracies for the classification of foot movement vs. rest. This difference might not fully explain all interactions found in the study, since only the interaction of sex with comparison was significant, but no other interaction with sex. However, we could assume that a better classification of imagery-related brain signals in men could at least partly explain the group effect.

In contrast to the presented classical BCI application with motor imagery and surface EEG, microelectrode arrays were used to catch intuitive command signals. It was shown that via microelectrode arrays implanted to the motor cortex, patients with tetraplegia can accurately control robotic aids up to 5 years after implantation (7). By combining invasive microelectrode arrays with neuroprosthetic limbs, natural and intuitive command signals for hand movement can be recovered, as shown in animal models (86) and also in humans (10, 87). These examples show that much more precise measurement such as obtained with microelectrode arrays opens the door to targeting brain signals that are much more reliable than voluntary brain activation, because the intuitive movement-related brain signals can be produced without any effort by the patients.

### 4.3. High-dimensional space classifications

Network measures represent a challenge for machine learning. The feature vectors are very long and the search for the optimal feature vector is necessary in order to avoid overfitting. The simplest solution to find the optimal feature vector is to minimize the true error using all possible combinations of features and all reasonable lengths of feature vectors and to validate each combination using samples which were not in the training data set (88). Considering the exponential complexity requiring 2^|*F*|^ iterations, with |*F*| being the dimension of the feature space, this approach is computationally not feasible, and huge amounts of data would be necessary. Therefore, in practice, an iteration over the feature entries depends very much on the ordering of the features, the number of available features, redundancy within this set of available features, and a stopping criterion when iteration over the whole vector takes too much time and could lead to undesirable overfitting.

We speculate that for the ffDTF already the low-density whole-head montage leads to poor performance of the implemented feature vector optimization. In addition, we might speculate that brain networks related to movement do not add any additional information to what is already contained in the power spectrum. It is possible that the redundancy of information prevents the feature vector from converging to an optimum comparable to the result obtained with classical power spectral data.

We need to consider that the feature subset selection technique we have employed was not powerful enough to tap the full potential of HD-EEG. In the literature, there were more sophisticated approaches to selecting a small subset of channels for BCIs, such as a sparse common spatial pattern algorithm which yielded an improvement of 10% in accuracy compared to the use of the low-density sensomotoric-montage (C3, C4, Cz) (46). Another approach to solve the small sample size problem arising from the misbalance between available training and test data in comparison to the high number of features was introduced by Meng et al. (89). The proposed method iteratively adds testing data as part of the training data with labels that were predicted in a previous iteration. This allows to increase the classification accuracy even in situations with small training sets of no more than 30 trials. Also in line with the high-dimensional classification problem we might have encountered, Meng et al. reported recently (90) that a BCI configured based on common spatial patterns and 40 channels showed no improvement over the configuration with 9 channels; moreover, a small configuration with 9 channels and laplacian filtering outperformed the other two configurations on the long run when subjects improved their skills of using the BCI over sessions.

### 4.4. Limitations

A major shortcoming in this study with respect to the comparison of patients with controls is the difference in age, which is considerable. Moreover, there might be differences in motor response and imagery abilities because of time since injury, type of injury, and impairment. Due to the small sample size, we can not address these issues, and suggest that future studies should be designed in order to determine the possibilities to use motor and imagery signals for control of a BCI in various subgroups of patients with SCI.

As stated in the methods, all conditions were accompanied by the pace-making sound, so that a differentiation by means of the acoustic response should not be possible. However, the result could be different without such a pace-maker, so that future investigations should consider comparing the experimental setup with and without pace-maker sound. We did not implement such a contrast because the experiment already took very long.

The fact that the higher frequency bands were prominently selected might either mean that there was indeed meaningful information in this bandwidth, or that the classification was disturbed by poor quality. In the latter case, the poor classification results might partly be explained by non-informative data in the highest frequency band, which were affected by noise.

The original number of trials, i.e., 25, is too low for performing any kind of machine learning. Therefore, we performed a simple type of data augmentation, by splitting the segments into 6 sub-segments. This results in sub-segments that are not completely independent from each other, so that we ensured that each 6 sub-segments of one trial fall into the same fold of cross validation. Other publications that work with deep learning use even less independent scenarios of data augmentation with EEG data (91). In such a scenario one would create 999 segments out of the 6 s by using a sliding window that shifts over the segment and creates 1 s segments starting from each sample point. Our approach is—to our opinion—a reasonable compromise between this complete loss of independency and perfect independency, in alignment with the need for a large sample size that can be used for training a classifier.

Moreover, the trials were all collected in a single session, so there is a considerable chance that the trials are similar to each other just as the subsegments of the trials are. A grouping by session with sessions conducted on different days would be highly recommendable (92) in future work.

The choice of the reference might be an advantage or disadvantage, since Cz as a reference enhances the motor-signal over all electrodes, whereas the activity over Cz is obtained in a bipolar manner, only. In clinical settings the bipolar reference is a standard for review, because it enhances the contrast. Since the purpose of this study was to examine different spatial configurations, we did not examine different reference settings. Future work should address this issue, because it might impact the results.

With respect to the statistical method we need to consider that we had an unbalanced sample size of 14 vs. 22 or 7 vs. 22 when analysing only the first recordings of the patient sample. Such a situation may affect the *p*-value, especially when the variances strongly differ between groups. Nevertheless, this applies mostly to the *p*-values which have marginally reached significance before correcting for multiple comparisons, such as 0.04, while the effects identified in this study with *p*-values < 0.001 seem plausible.

We examined the right hand and right foot, only, because examination of both, the left and the right side, would have doubled the duration of the task. The overall duration of the experiment was already quite long. With EGI's HD-EEG system the duration of an experiment is also limited by the nets, where sponges tend to dry out when an examination lasts longer than approximately 1.5 h, depending on the air moisture and temperature. Moreover, the task of performing motor imagery etc. is quite monotone and puts high demands on the attention of participants. These demands increase with the duration of the experiment. However, because of the given design, we cannot generalize our results to left side movements, where the activation patterns might be different.

The calculation of the ffDTF requires choosing a model order. Reliability of measures of interaction depends on the model order (57). With a given model order, a decreasing length of the signal negatively affects reliability. But on the other hand, with a given length of the signal, an increasing model order positively influences reliability. However, this is only true when the ratio between to be estimated coefficients and model order *N*/(*M*·*p*) is within a reasonable range. Thus, the model order cannot be increased without considering the length of the signal. The suggestion that this ratio should be at least one is the minimum requirement. Thus, the optimum for a reliable result depends most likely on the ratio between model order and length, but a practical demonstration of the sweet spot in this relation would require immense computing facilities. Therefore, we restricted ourselves to the maximum possible model order for each configuration. When restricting the analysis to the low-density montage we calculated the multivariate autoregressive model solely based on the respective channels, and thus, with a higher model order, as the number of channels beneficially adds to the ratio between samples and numbers of coefficients to be estimated for the multivariate autoregressive model. However, in doing so, we have a bias of model order, thus an advantage of the low-density configurations which is not related to spatial coverage but to the way the signal was processed. In view of the present results we could suggest the use of a larger number of trials. This would allow to increase the model order also for the high-density configuration, and possibly lead to even better results. However, since the acquisition procedure was already quite challenging for the patients, such a study can probably only be done in young healthy controls, and we have to leave this speculation open to further, technical investigations of the optimal model order for brain network parameters for BCIs.

The HD-EEG hardware used in this study is not comparable to low-density EEG hardware as used in other studies relying on low-density EEG, so that our results for the low-density configuration do not necessarily correspond to what other researchers have reported for low-density recordings. The sponges with electrolyte water represent a different recording technique than the classical Ag-AgCl electrodes alongside with abrasive electrolyte paste. The impedances recommended by the supplier of the HD-EEG system are well above the impedances typically achieved with classical EEG-systems, whereas the high signal to noise ratio should allow to still get high-quality data (93). These technical differences limit the translation of the results from our low-density configuration to classical 10–20 EEG systems. However, the HD-EEG system used in this study represents a technology that can be applied in a short amount of time, so that this is a major advantage when using it with patients. One possible solution would have been to perform two separate sessions with HD-EEG and classical 10–20 system EEG. Nevertheless, this poses additional challenges to the study design, such as expect learning effects, effects of the time of the day for the two separate recordings, or the day-to-day variance when performing the examinations on separate days.

In addition to the problematic day-to-day variance which is an inherent property of the EEG, the fact that our study was performed in a single session and in an off-line manner does not allow to assess the potential effect of learning that has been recently reported (94). In order to transfer any knowledge gained in off-line experiments, we would need to replicate the sessions, in order to demonstrate the stability of the extracted features and classification accuracies.

Finally, we did not exclude artifacts because of the low trial numbers in this study. After excluding artifacts the classification would have been unfeasible. Moreover, without rejection of artifacts the present analysis is closer to a real-time processing BCI scenario. Nevertheless, the advantage of ffDTF to be still reliable in artifact-contaminated data did not yield a higher accuracy than the FFT, which is more likely to be affected by artifacts. At the very least, the movement conditions could be thought to be affected by the artifacts, which is likely to be the case in the condition of foot movement in the control group where classification accuracies were quite high. The movement of the foot is more likely than the movement of the hand to cause small movements of the whole body, which could artificially lead to increased classification accuracies in all binary classification scenarios involving foot movement. In addition, it is possible that certain conditions evoked a higher rate of eye movement artifacts, which might have affected the result. The number of trials in this study was too low in order to exclude artifacts rigorously. Future studies with a lower number of conditions could increase the number of trials and remove ocular artifacts.

### 4.5. Conclusions and future directions

We claim that full-montage configurations may be recommendable for BCIs, regardless of whether a classical 10–20 system or HD-EEG is used. With HD-EEG, coverage of the sensorimotor region is as good as the whole scalp montage, and as good as a full-montage 10–20 system. The implication is, thus, that we do not need HD-EEG but full-montage EEG. This recommendation is not restricted to clinical populations, e.g., in order to improve the BCI performance in patients with SCI. Also healthy participants yielded higher classification accuracies with the full-montage configurations. The use of synchrony measures, concretely the ffDTF is not recommendable according to our data, because the long feature vectors prevented the feature subset selection algorithm from finding the relevant information in the feature vector. However, the comparison of HD-EEG to a downscaling to the 10–20 system in our work is not directly comparable to other studies using 10–20 systems, since there are also major hardware differences, so that further studies with recordings with different hardware are needed in order to directly compare HD-EEG with classical low-density montages. In addition, the study is limited to laboratory settings with a specific paradigm that involves only motor system activation in a synchronized design. The presented methods may fail in an asynchronous BCI, and since the system is optimized toward the motor system it may not work efficiently when multiple cortical systems are involved simultaneously. Thus, the question which montage is better suited might depend on the specific application.

Our results extended previous findings by using a larger frequency range, including also delta, theta, and gamma frequencies. We speculate that the slowing of motor-related activity to lower frequencies in patients with SCI could moderate BCI performance in this population. Analysis of lower frequencies could be a simple modification in order to handle this problem.

## Author contributions

YH designed the study, performed the analysis, wrote the first draft of the paper, revised it, and implemented all comments from the coauthors to create the final version. AT and PH performed preparation of study materials, recording of the data, recruitment of patients, and data management, and contributed to the technical part of the manuscript. AU and AB supervised the work in technical and statistical respects and contributed ideas to how the analysis should be performed and how the results should be presented. SL performed neurological examinations of the patients. SL, RN, and ET supervised the work in clinical respects. All of the listed authors have read, commented, and approved the manuscript.

### Conflict of interest statement

The authors declare that the research was conducted in the absence of any commercial or financial relationships that could be construed as a potential conflict of interest.
